# Comparative analysis of chemical constituents in Citri Exocarpium Rubrum, Citri Reticulatae Endocarpium Alba, and Citri Fructus Retinervus

**DOI:** 10.1002/fsn3.2897

**Published:** 2022-04-22

**Authors:** Wanling Yang, Mengshi Liu, Baizhong Chen, Jinrong Ning, Kanghui Wang, Yi Cai, Depo Yang, Guodong Zheng

**Affiliations:** ^1^ Guangzhou Municipal and Guangdong Provincial Key Laboratory of Molecular Target & Clinical Pharmacology, The NMPA and State Key Laboratory of Respiratory Disease, School of Pharmaceutical Sciences and the Fifth Affiliated Hospital Guangzhou Medical University Guangzhou China; ^2^ Guangdong Xinbaotang Biological Technology Co., Ltd Jiangmen China; ^3^ School of Pharmaceutical Sciences Sun Yat‐sen University Guangzhou China

**Keywords:** Citri Fructus Retinervus, Citri Exocarpium Rubrum, Citri Reticulatae Endocarpium Alba, GC–MS, HPLC–PDA, UHPLC–Q‐Exactive Orbitrap–MS

## Abstract

Citri Exocarpium Rubrum (CER), Citri Reticulatae Endocarpium Alba (CREA), and Citri Fructus Retinervus (CFR) are used as medicine and food, which derive from three different parts of the pericarp of *Citrus reticulata* Blanco through natural drying. To systematically investigate similarities and differences in phytochemicals about the three herbs, a series of analytic approaches were applied for the qualitative and quantitative analysis of chemical constituents in them. The results indicated a total of 48 volatile compounds were determined representing 99.92% of the total relative content of CER extracts, including 24 alkenes, 11 alcohols, 6 aldehydes, 2 ketones, and 2 phenols, while volatile compounds were not extracted from CREA and CFR. CER was abundant in volatile components that mainly existed in the oil gland. And a total of 32, 35, and 28 nonvolatile compounds were identified from CER, CREA, and CFR extracts, respectively. The total content of flavonoids and phenolic, and hesperidin in CFR was the highest, followed by CREA and CER. Conversely, CER was a rich source of polymethoxyflavones (PMFs), and the total polymethoxyflavone content (TPMFC), the content of nobiletin, 3,5,6,7,8,3′,4′‐heptamethoxyflavone (HMF), tangeretin, and 5‐hydroxy‐6,7,8,3′,4′‐pentamethoxyflavone (5‐HPMF) in CREA and CFR were extremely low. Besides, CER and CREA had a higher concentration of synephrine than CFR. The phytochemicals of CER, CREA, and CFR were significantly different, which might provide chemical evidence for the comparative pharmacological activities’ research and rational application of them.

## Graphical abstract

Comparative analysis of the chemical constituents in Citri Exocarpium Rubrum, Citri Reticulatae Endocarpium Alba, and Citri Fructus Retinervus.

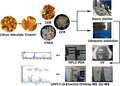

## 1 INTRODUCTION

As traditional Chinese medicines (TCMs), Citri Exocarpium Rubrum (CER), Citri Reticulatae Endocarpium Alba (CREA), and Citri Fructus Retinervus (CFR) derive from three different parts of the pericarp of *Citrus reticulata* Blanco through natural drying. CER, the dried outer pericarp of *Citrus reticulata* Blanco and its cultivars, known as “Juhong” in China, is widely used to eliminate dampness and phlegm, and other respiratory diseases (Pharmacopoeia CON, 2020). CREA, the dried middle pericarp of *Citrus reticulata* Blanco and its cultivars, is employed to improve digestion by strengthening spleen function (Chinese Herbalism Editorial Board, 1999) and its Chinese name is “Jubai.” CFR, called “Juluo” in China, the endothecium ligamentum of the fruit peel of *Citrus reticulata* Blanco and its cultivars, is traditionally applied to dredge meridian and promote blood flow (Chinese Herbalism Editorial Board, 1999).

CER, CREA, and CFR have been widely applied in clinical areas to treat different diseases based on the theory of traditional Chinese medicine (TCM), which has been considered to be related to the chemical profiling and the concentration of bioactive constituents. At present, research on the three TCMs mainly concentrated on their pharmacological activities (Liu et al., 2008; Wang et al., 2016; Xiao et al., 2009). For instance, the extracts of five citrus herbs (CitriReticulataePericarpium [CRP], CitriExocarpium Rubrum [CER], Citri Grandis Exocarpium [CGE], Aurantii Fructus Immaturus [AFI], and Aurantii Fructus [AF]) showed inhibitory effects on acetylcholinesterase and α‐glucosidase in a concentration‐dependent manner (Guo et al., 2021). Regarding the chemical profiling of them, a few studies focused on flavonoids, such as hesperidin and nobiletin (Zhao et al., 2017). However, there is a lack of research systematically and comparatively reporting on the chemical constituents of the three herbs.

The Citrus reticulata “Chachi” pericarp (CRCP), from Sanjiang of the Xinhui region (Guangdong Province, China), is one cultivar of Citri Reticulatae Pericarpium (CRP) and the main plant materials of CRCP. According to the previous studies (Zheng, Zeng, et al., 2019), CRCP, particularly planted and harvested in the Xinhui region (Guangdong Province, China), is traditionally considered to have superior qualities compared with RCP of other varieties. Therefore, *Citrus reticulata* “Chachi” was selected as the materials of CER, CREA, and CFR in the study, since it can be used as an excellent source of *Citrus reticulata* Blanco in clinical application and by‐product development.

Owing to the complicated compositions and various natural medicinal plants, a combination of analytical techniques is required to analyze their phytochemical compositions (Choe et al., 2020). For example, combining the separation ability of chromatography with the qualitative function of mass spectrometry, ultra‐high performance liquid chromatography combined with quadrupole‐Exactive Orbitrap tandem–mass spectrometry (UHPLC–Q‐Exactive Orbitrap–MS/MS) technology is widely used for rapid analysis of plant extracts with higher separation efficiency, higher throughput, faster scanning speed, and higher sensitivity than high performance liquid chromatography–mass spectrometry (HPLC–MS) (Zheng, Li, et al., 2020).

Thus, a series of analytic approaches were employed to comprehensively determine chemical components of the three herbs in this study. Gas chromatography–mass spectrometry (GC–MS) was used for the analysis of volatile components first. Simultaneously, UHPLC–Q‐Exactive Orbitrap–MS analysis was used for the qualitative analysis of differences in nonvolatile constituents. Furthermore, the contents of important nonvolatile compounds, including flavanone glycosides, PMFs, phenolic and synephrine, were determined and compared by ultraviolet (UV) and high‐performance liquid chromatography–photodiode array detection (HPLC–PDA). It is conducive to further clarify the differences in the chemical constituents of CER, CREA, and CFR, which may provide the scientific basis for explaining the different pharmacological activities and rational development of them.

## 2 MATERIALS AND METHODS

### 2.1 Plant materials

About 20 kg *Citrus reticulata* “Chachi” were gathered in December, 2020 from Sanjiang of the Xinhui region (Guangdong, China), which were certified by Prof. Guodong Zheng. According to the botanical characteristics of *Citrus reticulata* Blanco, samples of fruit peel of *Citrus reticulata* “Chachi” were separated into the outer peel, the middle outer peel, and the endothecium ligamentum, which became CER, CREA, and CFR, respectively, after drying and were kept in the Laboratory of Pharmacognosy, Guangzhou Medical University, Guangdong Province, China.

### 2.2 Chemical materials

The solvents, acetonitrile and methanol of HPLC‐grade, were obtained from Merck. Chromatographic‐grade formic acid and hexane were purchased from Thermo Fisher Scientific and Honeywell , respectively. Ultrapure water was available using a Milli‐Q system (Millipore). The other reagents were of analytical grade and purity and commercially available. The reference standards (gallic acid, synephrine, hesperidin, nobiletin, tangeretin, ferulic acid, narirutin, and limonin) were obtained from Must Biotechnology. The other reference standards (3,5,6,7,8,3′,4′‐heptamethoxyflavone (HMF), isoscopoletin, scopoletin, scoparone, stachydrine) were purchased from Weikeqi Biological Technology Co., Ltd. and 5‐hydroxy‐6,7,8,3′,4′‐pentamethoxyflavone (5‐HPMF) was obtained from Spring & Autumn Biological Engineering Co., Ltd. The purity of these reference standards was above 98%.

### 2.3 Sample preparation

Approximately 100 g of CER, CREA, and CFR was used for the extraction of volatile compounds according to the method described in the 2020 edition of Chinese Pharmacopoeia (Pharmacopoeia, 2020). A 50 μL aliquot of the volatile compounds was dissolved in 950 μL of hexane solvent and filtered through a 0.22‐μm membrane before GC–MS analysis.

The samples were ground into powder and passed through a 40‐mesh sieve. Each dried sample powder (0.2 g) was weighed accurately and was extracted by ultrasonic treatment in a KQ‐800KDE instrument (Kunshan Ultrasonic Instruments Co. Ltd) with 20 ml methanol for 30 min at 320 W (40 kHz) and an aliquot of 1 μl of the filtrate was injected for UHPLC–Q‐Exactive Orbitrap–MS analysis.

Similarly, each sample powder (0.2 g) was extracted by ultrasonic treatment with 20 ml methanol or ethyl acetate and then filtered to obtain the sample solution. The above methanol extracts were prepared for the determination of the total flavonoid content (TFC) and the total phenolic content (TPC), and the ethyl acetate extracts were prepared for the determination of the total polymethoxyflavone content (TPMFC). Methanol extracts (1 ml) were filtered through a 0.22‐μm membrane before the HPLC–PDA analysis.

### 2.4 GC–MS analysis system for volatile components

GC–MS analysis was carried out with an Agilent 7890A gas chromatography system equipped with Agilent DB‐5MS Ultra Inert capillary GC column (30 m × 0.25 mm, 0.25 μm) and a 5975C mass spectrometer equipped with a triple‐axis detector (Agilent). The heating program settings were as follows: (i) the temperature was set at 60°C first and then increased to 80°C at a rate of 1°C·min^−1^ for 10 min; (ii) the temperature was ramped up to 250°C at a rate of 5°C·min^−1^ and to 300°C at 20°C·min^−1^ for 1 min finally. The other procedures were set as follows: electron impact (EI^+^) mode: 70 eV; detector temperature: 270°C; injection volume: 5 μL; injection port temperature: 270°C; high‐purity helium flow rate: 1 ml·min^−1^; split ratio: 10:1; scan speed: 0.2 amu·s^−1^ (from *m/z* 30 to 550 amu); solvent delay: 4 min. All volatile components were determined by comparing the mass spectra with the NIST08.L database.

### 2.5 UHPLC–Q‐Exactive Orbitrap–MS analysis system for nonvolatile compounds

The extracts samples were separated on a ZORBAX SB‐C_18_ column (4.6 mm × 50 mm, 1.8 μm) with a flow velocity of 0.40 ml·min^‐1^ at 40°C. The mobile phase was composed of 0.1% (v/v) formic acid solution (phase A) and acetonitrile (phase B). Gradient elution system was as follows: 0–2 min, 5%–5% B; 2–4 min, 5%–25% B; 4–6 min, 25%–25% B; 6–10 min, 25%–50% B; 10–14 min, 50%–85%; 14–16 min, 85%–90%.

The conditions of the MS system were set as follows: the ion spray voltage was maintained at 3.5 kV. The auxiliary gas heater and capillary temperatures were retained at 300 and 320°C, respectively. The sweep gas, auxiliary gas, and sheath gas were, respectively, sustained at 1.7, 3.3, and 10.0 L min^−1^. Chemical compound data were gathered from *m/z* 70 to 1000 Da in the full MS scan mode with a resolution of 70,000.

### 2.6 UV analysis for the determination of TFC, TPMFC. and TPC

TFC and TPMFC were determined using the external standard method with hesperidin and nobiletin. The methanol extracts for the determination of TFC and the ethyl acetate extracts for the determination of TPMFC were diluted to a proper concentration and the absorbance was measured using a UV 2600 instrument (Shimadzu) at 283 and 330 nm, respectively.

TPC was calculated using the folin phenol colorimetric assay. Methanol extracts (0.1 ml) mixed with folin phenol reagent (1 ml) and ultrapure water (0.4 ml) were kept in the dark for 2 min. Then, ultrapure water (1 ml) and Na_2_CO_3_ (7.5% w/v, 2 ml) were added. The above‐mixed solution was allowed to react in the dark at 40°C for 30 min. The absorbance of the mixed solution was measured at 765 nm with gallic acid as a reference standard.

### 2.7 HPLC–PDA analysis system for the determination of six bioactive components

A methanol mixture of six standard substances was diluted for obtaining various concentrations to establish calibration curves. The six standard substances included hesperidin (283 nm), nobiletin (330 nm), HMF (330 nm), tangeretin (330 nm), 5‐HPMF (330 nm), and synephrine (224 nm). The extracts samples were separated on a Diamonsil C_18_ column (250 mm × 4.6 mm, 5 µm) and the mobile phase consisted of 0.1% (v/v) phosphoric acid water solution (phase A, pH 3.70) and 50% methanol +50% acetonitrile (phase B). The gradient elution condition was as follows: 0–5 min, 5%–5% B; 5–10 min, 5%–55% B; 10–15 min, 55%–60% B; 15–20 min, 60%–65%; 20–25 min, 65%–75%; 25–30 min, 75%‐–80% B; 30–35 min, 80%–85% B; and 35–40 min, 85%–90% B. The flow rate was set at 1 ml·min^‐1^ and the injected sample volume was 10 μl.

## 3 RESULTS

### 3.1 GC–MS analysis of volatile compounds in CER

The extraction yield of volatile compounds in CER in this study was 5.44%, while almost no volatile compounds were extracted from CREA and CFR. A total of 48 volatile compounds were determined which accounted for 99.92% of all identified volatile compounds, which also mean the relative percentage of 48 volatile compounds representing 99.92% of the total relative content of CER volatile extracts. The 48 volatile compounds included 24 alkenes, 11 alcohols, 6 aldehydes, 2 ketones, 2 phenols, and others, of which the bioactive components mainly were D‐limonene (71.71%), γ‐terpinene (11.97%), α‐pinene (2.71%), β‐pinene (2.01%), myrcene (2.71%), methyl 2‐(methylamino)benzoate (1.98%), isopropyltoluene (1.31%), terpinolene (0.95%), and so on (Table 1).

**TABLE 1:** Forty‐eight volatile compounds identified in Citri Exocarpium Rubrum (CER) by gas chromatography–mass spectrometry (GC‐MS)

No.	t_R_(min)	Compound formula	Identified compound	Relative percentage (%)
Alkenes				
1	6.96	C_10_H_16_	Sabinene	0.93
2	7.27	C_10_H_16_	α‐Pinene	2.71
3	7.97	C_10_H_16_	Camphene	0.03
4	9.07	C_10_H_16_	Sabinene	0.27
5	9.33	C_10_H_16_	β‐Pinene	2.01
6	10.00	C_10_H_16_	Myrcene	2.71
7	10.93	C_10_H_16_	α‐Phellandrene	0.11
8	11.68	C_10_H_16_	α‐Terpinene	0.30
9	12.05	C_10_H_14_	p‐Cymene	1.31
10	13.01	C_10_H_16_	D‐Limonene	71.71
11	13.74	C_10_H_16_	3‐Carene	0.02
12	14.71	C_10_H_16_	γ‐Terpinene	11.97
13	16.59	C_10_H_16_	Terpinolene	0.95
14	16.96	C_10_H_12_	p‐Cymenene	0.02
17	18.87	C_10_H_14_	1,3,8‐p‐Menthatriene	0.05
19	19.92	C_10_H_18_O	Bicyclo[3.1.0]hexan‐2‐ol, 2‐methyl‐5‐(1‐methylethyl)‐	0.01
23	24.41	C_10_H_16_	2‐Carene	0.01
38	40.94	C_15_H_24_	Copaene	0.03
39	41.45	C_15_H_24_	Cubebene	0.03
42	42.53	C_15_H_24_	Caryophyllene	0.08
43	45.02	C_15_H_24_	α‐Selinene	0.03
44	45.36	C_15_H_24_	Farnesene	0.17
45	45.69	C_15_H_24_	Cadinenes	0.03
46	47.42	C_15_H_24_O	Caryophyllene oxide	0.01
Alcohols				
15	17.97	C_10_H_18_O	Linalool	0.13
18	19.71	C_10_H_16_O	2‐Cyclohexen‐1‐ol,1‐methyl‐4‐(1‐methylethenyl)‐	0.08
20	21.12	C_10_H_16_O	cis‐p‐Mentha‐2,8‐dien‐1‐ol	0.08
21	22.49	C_10_H_18_O	Cyclohexanol,1‐methyl‐4‐(1‐methylethenyl)‐	0.02
24	26.13	C_10_H_18_O	L‐Terpinen‐4‐ol	0.34
25	27.20	C_10_H_14_O	p‐Cymenol	0.08
26	28.32	C_10_H_18_O	α‐Terpineol	0.40
27	28.97	C_10_H_16_O	Carveol	0.12
33	37.16	C_10_H_16_O	2‐(4‐methylidenecyclohexyl)prop‐2‐en‐1‐ol	0.02
35	37.59	C_10_H_16_O	1‐Perillyl alcohol	0.01
37	40.52	C_10_H_18_O	Nerol	0.01
Aldehydes				
16	18.43	C_9_H_18_O	Nonanal	0.02
22	23.12	C_10_H_18_O	6‐Octenal, 3,7‐dimethyl‐	0.03
28	30.73	C_10_H_20_O	Decanal	0.08
31	36.08	C_10_H_14_O	Perillaldehyde	0.06
41	42.41	C_12_H_24_O	Dodecanal	0.02
47	51.49	C_15_H_22_O	(E,E,E)‐2,6,10‐trimethyldodeca‐2,6,9,11‐tetraen‐1‐al	0.24
Ketones				
29	31.73	C_10_H_14_O	Carvone	0.49
36	38.07	C_9_H_10_O_2_	1‐(2‐hydroxy‐5‐methylphenyl)‐ethanone	0.01
Phenols				
32	36.90	C_10_H_14_O	Thymol	0.01
34	37.35	C_10_H_14_O	Phenol, 2‐methyl‐5‐(1‐methylethyl)‐	0.13
Others				
30	32.24	C_10_H_16_O	2‐Carene epoxide	0.02
40	42.11	C_9_H_11_NO_2_	Methyl 2‐(methylamino)benzoate	1.98
48	55.93	C_16_H_32_O_2_	Palmitic acid	0.04

### 3.2 Analysis of differences in the nonvolatile chemical composition of CER, CREA, and CFR

**FIGURE 1:**
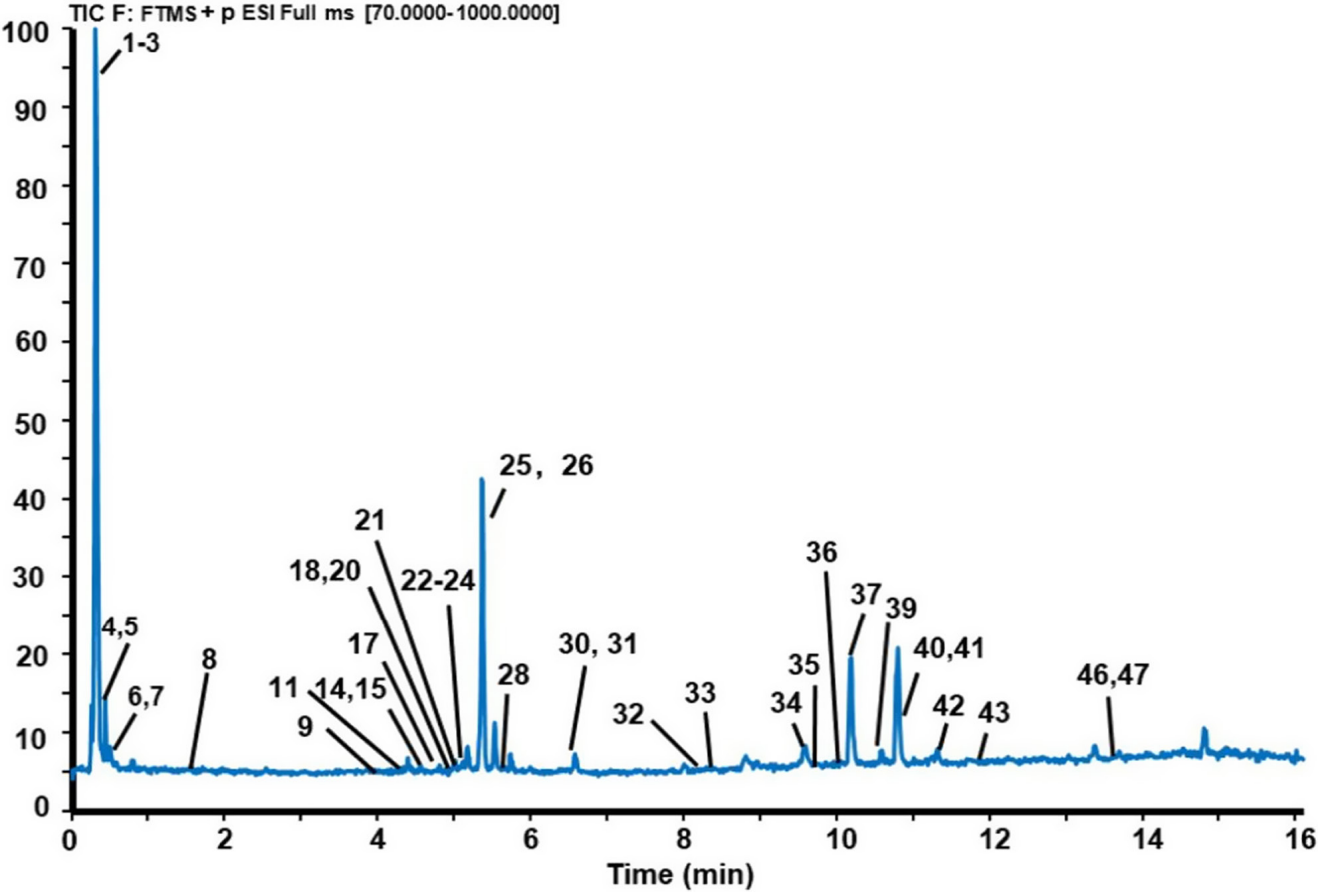
The total ion chromatogram (TIC) of Citri Reticulatae Endocarpium Alba (CREA) in positive model

**FIGURE 2:**
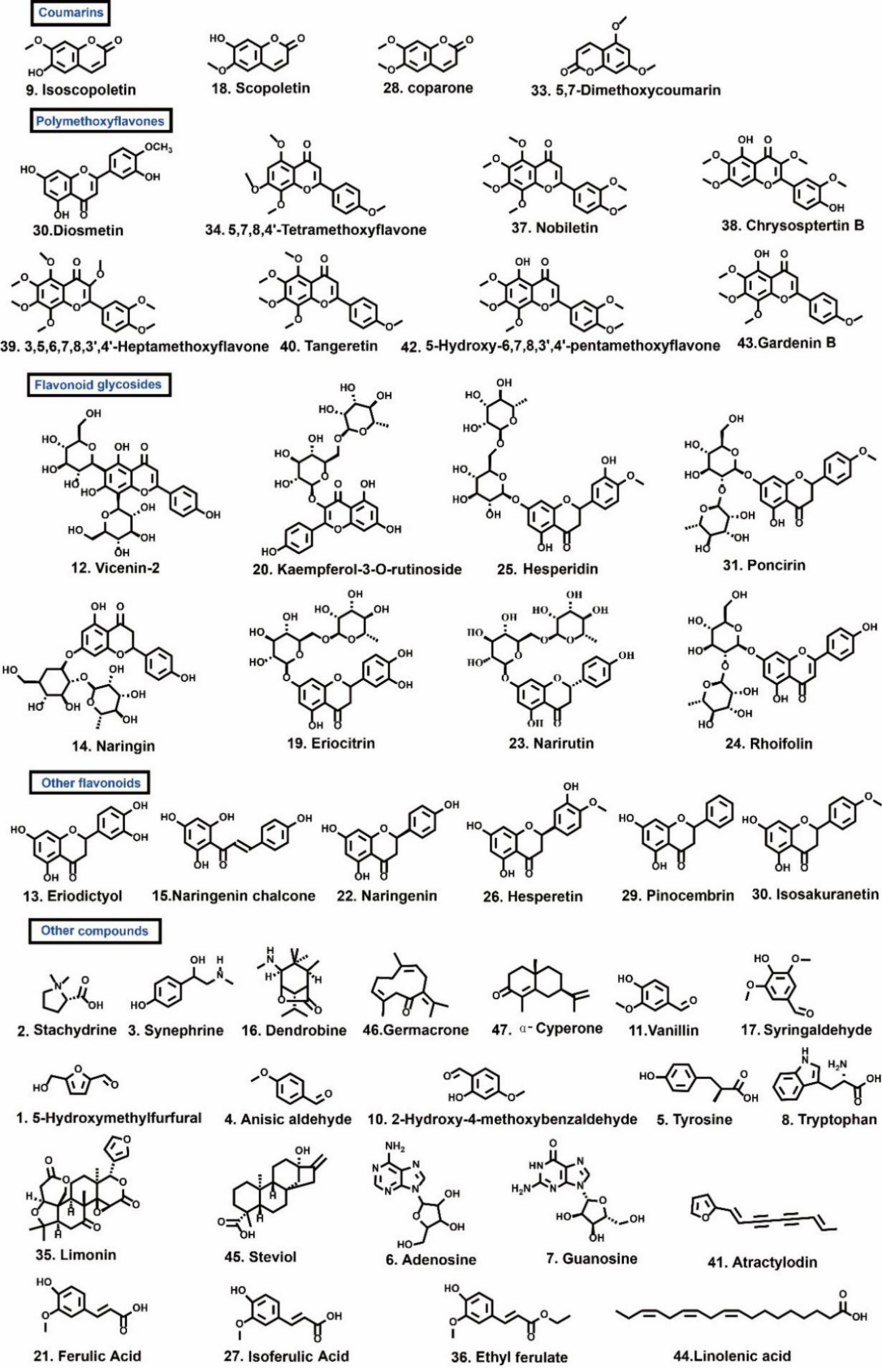
Chemical structures of 47 nonvolatile compounds identified by ultra high performance liquid chromatography–quadrupole Exactive Orbitrap–mass spectrometry (UHPLC–Q‐Exactive Orbitrap–MS)

By optimizing a series of parameters such as the elution gradient of mobile phase and flow rate, better analytical conditions were obtained and were described in “Section 2.5.” The total ion chromatogram (TIC) of CREA in the positive model is shown in Figure 1. According to the retention time and fragment ions’ information of constituents provided by the UHPLC–Q‐Exactive Orbitrap–MS analysis, a total of 47 compounds (Table 2) were separated within 16 min and identified by comparing with fragment ions provided by the Orbitrap Traditional Chinese Medicine Library (OTCML), relative literature, and standard substances, including 4 coumarins, 8 PMFs, 8 flavonoid glycosides (5 flavanone glycosides), 6 other flavonoids, and 21 other compounds (3 alkaloids, 2 terpenoids, 5 organic acids, 3 aldehydes, 1 limonoid, and so on). All of these chemical structures are displayed in Figure 2.

**TABLE 2:** Nonvolatile compounds identified in Citri Exocarpium Rubrum (CER), Citri Reticulatae Endocarpium Alba (CREA), and Citri Fructus Retinervus (CFR) by ultrahigh performance liquid chromatography–quadrupole Exactive Orbitrap–mass spectrometry (UHPLC–Q‐Exactive Orbitrap–MS)

No.	*t*_R_ (min)	Experimental [M + H]^+^(*m/z*)	Major secondary fragment ions (*m/z*)	Compound formula	Identification	CER	CREA	CFR	Ref/standard
Coumarins									
9	3.93	193.0498	178.0261, 165.0546, 149.0598,143.0598, 137.0597, 133.0285, 122.0365, 115.0544, 105.0702, 91.0546, 79.0579	C_10_H_8_O_4_	Isoscopoletin	√	√		^a^
18	4.86	193.0498	178.0263, 165.0549, 150.0313, 137.0598, 133.0286, 122.0365, 117.0336, 105.0341, 89.0391, 77.0393, 66.0471, 53.0395	C_10_H_8_O_4_	Scopoletin	√	√		Zeng et al. (2015)^a^
28	5.63	207.0653	191.0339, 179.0703, 163.0390, 151.0754, 148.0519, 136.0519, 121.0650, 107.0495, 91.0547	C_11_H_10_O_4_	Scoparone	√	√		Duan et al. (2014)^a^
33	8.36	207.0653	192.0416, 189.0914, 179.0699, 164.0468, 151.0754, 148.0519, 133.0651, 121.0650, 118.0416, 91.0548, 67.0549	C_11_H_10_O_4_	5,7‐Dimethoxycoumarin		√		Duan et al. (2014)^a^
Polymethoxyflavones									
32	8.20	301.0707	286.0472, 258.0523, 229.0498, 195.6004, 153.0181	C_16_H_12_O_6_	Diosmetin	√			Chen et al. (2019)
34	9.57	343.1174	328.0935, 313.0701, 299.0908, 285.0752, 270.0516, 257.0802, 199.0234, 181.0129, 153.0180, 133.0647	C_19_H_18_O_6_	5,7,8,4'‐Tetramethoxyflavone	√	√	√	Duan et al. (2014)
37	10.17	403.1387	388.1149, 373.0916, 358.0679, 327.0859, 313.0705, 301.0703, 284.0678, 258.0520, 244.0728, 229.0340, 211.0236, 183.0288, 165.0545	C_21_H_22_O_8_	Nobiletin	√	√	√	^a^
38	10.24	375.1075	360.0836, 345.0603, 327.0497, 317.0657, 271.0603 227.0557, 215.0185, 197.0080, 149.0598, 113.0234, 85.0290, 55.0184	C_19_H_18_O_8_	Chrysosptertin B	√			Zheng, Liu, et al. (2020)
39	10.58	433.1493	418.1257, 403.1023, 385.0915, 360.0839, 345.0603, 339.0510, 317.0863, 289.0704, 271.0593, 243.0867, 211.0239, 183.0289, 165.0547, 151.0744, 127.0381	C_22_H_24_O_9_	3,5,6,7,8,3’,4’‐Heptamethoxyflavone	√	√	√	^a^
40	10.78	373.1282	358.1042, 343.0812, 328.0581, 315.0853, 297.0759, 283.0604, 271.0603, 254.0574, 229.0320, 211.0237, 183.0289, 135.0422	C_20_H_20_O_7_	Tangeretin	√	√	√	^a^
42	11.31	389.1230	374.0992, 359.0757, 341.0652, 331.0807, 316.0574, 311.0511, 285.0749, 244.0728, 227.0546, 215.0184, 197.0080, 189.0545, 169.0131, 163.0752, 148.0518, 141.0181, 113.0236, 85.0289	C_20_H_20_O_8_	5‐Hydroxy‐6,7,8,3’,4’‐pentamethoxyflavone	√	√	√	^a^
43	11.89	359.1124	344.0891, 329.0656, 311.0549, 301.0705, 286.0471, 258.0526, 245.0810, 215.1826, 197.0086, 179.0345, 169.0131	C_19_H_18_O_7_	Gardenin B	√	√		Zheng, Liu, et al. (2020)
Flavonoid glycosides									
12	4.45	595.1660	577.1555, 559.1448, 541.1343, 511.1237, 481.1131, 457.1129, 409.0918, 379.0811337.0705, 325.0706, 295.0601, 283.0612, 268.0726, 121.0286	C_27_H_30_O_15_	Vicenin‐2	√			(Zeng et al., 2015)
14	4.55	581.1863	527.1508, 473.0620, 435.1271, 419.1349, 401.1240, 383.1123, 365.1022, 339.0856, 315.0864, 297.0763, 273.0756, 263.0546, 219.0285, 195.0288, 171.0287, 153.0182, 129.0547, 119.0494, 85.0290, 71.0499	C_27_H_35_O_14_	Naringin		√	√	^a^
19	4.89	597.1815	451.1240, 435.1270, 417.1201, 399.1068, 385.0961, 331.0811, 301.0726, 289.0707, 263.0551, 219.0295, 171.0288, 153.0391, 129.0547,85.0290, 71.0404	C_27_H_32_O_15_	Eriocitrin			√	Duan et al. (2014)
20	4.94	595.1658	449.1017, 403.4498, 343.0804, 287.0549, 161.0232, 153.0182, 85.0290	C_27_H_30_O_15_	Kaempferol‐3‐O‐rutinoside		√	√	Zheng, Liu, et al. (2020)
23	5.17	581.1863	545.9829, 435.1239, 419. 1336, 399.1061, 383.1125, 365.1010, 339.0860, 273.0757, 263.0549, 219.0288, 195.0289, 171.0288, 153.0182, 147.0441, 129.0547, 119.0494, 85.0290, 71.0499	C_27_H_35_O_14_	Narirutin		√	√	^a^
24	5.18	579.1709	433.1127, 271.0599, 247.0595, 171.0289, 153.0182, 119.0493, 85.0289, 71.0498	C_27_H_30_O_14_	Rhoifolin		√		Duan et al. (2014)
25	5.36	611.1969	303.0862, 263.0549, 219.0288, 195.0288, 177.0546, 153.0183, 129.0547, 85.0290, 71.0499	C_28_H_34_O_15_	Hesperidin	√	√	√	Duan et al., (2014)^a^
31	6.58	595.2023	518.8385, 449.1440, 433.1492, 415.1380, 397.1281, 379.1174, 353.1019, 311.0912, 287.0913, 263.0549, 219.028, 195.0289, 171.0288, 161.0597, 153.0183, 129.0547, 111.0443, 85.0290	C_28_H_34_O_14_	Poncirin	√	√	√	Duan et al. (2014)
Other flavonoids									
13	4.52	289.0707	271.0605, 225.0547, 182.9876, 179.0341, 171.0289, 163.0391, 153.01842, 135.0442, 119.0493,105.0702, 89.0391, 67.0186	C_15_H_12_O_6_	Eriodictyol			√	Xia et al. (2019)
15	4.56	273.0757	248.4177, 231.0658, 218.0436, 194.0842, 179.0347, 171.0289, 153.0184, 147.0442, 123.0445, 119.0496, 91.0548, 68.9978	C_15_H_12_O_5_	Naringenin chalcone	√	√	√	Yoshimura et al. (2009)
22	5.16	273.0757	255.0651, 231.0649, 218.0448, 194.0842, 179.0339, 153.0183, 147.0441, 129.0185, 119.0494, 107.0495, 91.0547, 68.9978	C_15_H_12_O_5_	Naringenin	√	√	√	Duan et al. (2014)
26	5.36	303.0862	285.0775, 261.0755, 179.0339, 177.0546, 171.0288, 163.0388, 153.0183, 149.0597, 135.0439, 123.0439, 117.0337, 89.0390	C_16_H_14_O_6_	Hesperetin	√	√	√	Duan et al. (2014)
29	6.39	257.0806	215.0703, 201.0464, 179.0340, 173.0595, 171.0287, 153.0182, 131.0491, 107.0494, 103.0545, 91.0547,	C_15_H_12_O_4_	Pinocembrin			√	Suleman et al. (2015)
30	6.58	287.0914	269.0811, 245.0808, 230.7657, 179.0340, 171.0289, 161.0598, 153.0183, 133.0649, 121.0650, 67.0186	C_16_H_14_O_5_	Isosakuranetin	√	√	√	Zheng, Liu, et al. (2020)
Other compounds									
1	0.30	127.0392	109.0285, 99.0442, 85.0652, 81.0343, 71.0499, 53.0394	C_6_H_6_O_3_	5‐Hydroxymethylfurfural	√	√	√	Zheng, Liu, et al. (2020)
2	0.31	144.1020	98.0968, 84.0814, 70.0658, 55.0550	C_7_H_13_NO_2_	Stachydrine	√	√	√	Zheng, Liu, et al. (2020)a
3	0.33	168.1020	150.0915, 135.0680, 121.0651, 119.0494, 111.2144, 107.0494, 91.0548	C_9_H_13_NO_2_	Synephrine	√	√	√	^a^
4	0.43	137.0599	122.3756, 119.04966, 109.0651, 95.0491, 94.0418, 93.0704, 91.0547, 81.0705, 79.0549, 67.0548, 53.0393	C_8_H_8_O_2_	Anisic aldehyde		√		Zheng, Yang, et al. (2020)
5	0.44	182.0814	165.0548, 147.0442, 136.0759, 123.0443, 119.0495, 95.0496, 91.0548	C_9_H_11_NO_3_	Tyrosine		√	√	Fuertig et al. (2016)
6	0.49	268.1041	136.0618, 119.0353, 94.0404, 85.0289, 57.0343	C_10_H_13_N_5_O_4_	Adenosine		√		Jimmerson et al. (2017)
7	0.50	284.0992	152.0569, 145.0495, 128.0457, 110.0352, 85.0290, 69.0341	C_10_H_13_N_5_O	Guanosine	√	√		Jimmerson et al. (2017)
8	1.54	205.0973	188.0706, 170.0602, 159.0918, 146.0601, 143.0730, 132.0809, 128.9509, 118.0654, 103.0548, 91.0546, 74.0244	C_11_H_12_N_2_O_2_	Tryptophan	√	√	√	Fuertig et al. (2016)
10	4.37	153.0549	145.1931, 135.0444, 129.9791, 125.0600, 111.0445, 109.0653, 100.5105, 93.0704, 88.9530, 70.9426, 65.0394	C_15_H_22_O	2‐Hydroxy‐4‐methoxybenzaldehyde			√	Fuertig et al. (2016)
11	4.38	153.0549	129.9789, 125.0595, 111.0439, 107.0854, 93.0338, 88.9527, 70.9424, 65.0393	C_8_H_8_O_3_	Vanillin	√	√		Fu et al. (2020)
16	4.57	264.1960	236.2011, 218.1908, 176.1433, 145.1013, 119.0859, 95.0859, 70.0659	C_16_H_25_NO_2_	Dendrobine	√			Wang, Wu, et al. (2016)
17	4.72	183.0654	159.9691, 155.0701, 140.0467, 131.9742, 123.0441, 113.9638, 105.0450, 95.0495, 90.9481, 81.0340, 72.9377, 65.0393	C_9_H_10_O_4_	Syringaldehyde	√	√		Fu et al. (2020)
21	5.00	195.0653	177.0547, 163.0390, 149.0598, 145.0285, 134.0363, 117.0338, 91.0548, 89.0391, 71.9409	C_10_H_10_O_4_	Ferulic acid	√	√	√	^a^
27	5.51	195.0653	177.0547, 163.0393, 149.0598, 145.0285, 134.0363, 117.0338, 91.0547, 89.0391, 79.0547	C_10_H_10_O_4_	Isoferulic acid	√			Shen et al. (2019)
35	9.69	471.2013	427.2109, 425.1955, 367.1902, 213.0909, 187.0753, 161.0597, 133.0648, 105.0702, 95.0132	C_26_H_30_O_8_	Limonin	√	√		^a^
36	10.03	223.0965	199.9964, 195.0920, 177.0546, 149.0234, 144.6860, 121.0287, 107.0859, 93.0703, 81.0704, 65.0393	C_12_H_14_O_4_	Ethyl ferulate		√	√	Zheng, Yang, et al. (2020)
41	10.83	183.0806	159.9691, 141.9588, 131.9743, 118.9676, 113.9639, 105.03387, 90.9482, 72.9378, 67.0549, 56.9430	C_13_H_10_O	Atractylodin			√	Zheng, Yang, et al. (2020)
44	12.71	279.2317	261.2214, 243.2099, 223.1695, 219.0568, 149.0232, 135.1170, 123.1169, 109.1014, 95.0859, 81.0704, 67.0549,	C_18_H_30_O_2_	Linolenic acid	√		√	Hu et al. (2018)
45	12.79	319.2265	301.2161, 273.2215, 255.2105, 217.1586, 165.1274, 147.1169, 137.1324, 119.0856, 109.1014, 95.0860, 81.0705, 69.0706	C_20_H_30_O_3_	Steviol	√		√	Molina‐Calle et al. (2017)
46	13.67	219.1745	201.1639, 191.1797, 177.1277, 173.1328, 159.1171, 149.0964, 145.1011, 135.1170, 131.0856, 121.1014, 117.0702, 107.0859, 93.0704, 81.0705, 71.0499	C_15_H_22_O	Germacrone		√		Fu et al. (2020)
47	13.68	219.1743	201.1637, 173.1327, 164.3430, 159.1168, 145.1011, 135.1169, 111.0809, 107.0858, 93.0703, 91.0578, 81.0705, 71.0498	C_15_H_22_O	α‐Cyperone	√	√		Fu et al. (2020)

^a^ Confirmation in the comparison with standard substances

A total of 32 compounds were identified from CER methanol extracts, including 3 coumarins, 8 PMFs, 3 flavonoid glycosides, 4 other flavonoids, and 14 other compounds. Simultaneously, 35 compounds (4 coumarins, 6 PMFs, 6 flavonoid glycosides, 4 other flavonoids, and 15 other compounds) were determined from CREA extracts and 28 compounds (5 PMFs, 6 flavonoid glycosides, 6 other flavonoids, and 11 other compounds) were identified from CFR. In general, 32, 35, and 28 nonvolatile compounds were, respectively, identified from CER, CREA, and CFR.

A total of 32, 35, and 28 nonvolatile compounds were, respectively, identified from CER, CREA, and CFR. It was worth noting that scopoletin, isoscopoletin, and scoparone were present in CER and CREA, and 5,7‐dimethoxycoumarin was exclusively found in CREA. While, the four coumarins were not found in CFR. Besides, diosmetin, chrysosptertin B, vicenin‐2, dendrobine, and isoferulic acid were exclusively present in CER. Rhoifolin, germacrone, anisic aldehyde, and adenosine were found only in CREA, while eriocitrin, eriodictyol, pinocembrin, 2‐hydroxy‐4‐methoxybenzaldehyde, and atractylodin were detected merely in CFR.

### 3.3 Determination of TFC, TPMFC, and TPC by UV

Flavonoids are considered as the primary bioactive constituents in citrus species, including flavonoid glycosides (flavonoid O‐glycoside or flavonoid C‐glycoside) and PMFs. As shown in Table 3, CER, CREA, and CFR were abundant in flavonoids, of which the total flavonoid content (TFC) of CFR was the highest (130.08 ± 0.11 mg g^−1^), followed by CREA (104.73 ± 0.00 mg g^−1^) and CER (89.23 ± 0.00 mg g^−1^). PMFs are special flavonoids that are mainly found in citrus. As seen in Table 3, the total polymethoxyflavone content (TPMFC) of CER was 10.78 ± 0.00 mg g^−1^, while those of CREA and CFR were extremely low. In addition, the total phenolic content (TPC) of CER, CREA, and CFR, respectively, was 26.73 ± 0.00 mg g^−1^, 40.67 ± 0.00 mg g^−1^, and 48.47 ± 0.08 mg g^−1^.

**TABLE 3:** The results of the determination of nonvolatile constituents in Citri Exocarpium Rubrum (CER), Citri Reticulatae Endocarpium Alba (CREA), and Citri Fructus Retinervus (CFR) by ultraviolet (UV) and high‐performance liquid chromatography–photodiode array detection (HPLC‐PDA)

Sample	UV (mg·g^−1^)^a^			HPLC–PDA (mg·g^−1^)^a^					
	TFC	TPMFC	TPC	Hesperidin	Nobiletin	HMF	Tangeretin	5‐HPMF	Synephrine
CER	89.23 ± 0.00	10.78 ± 0.00	26.73 ± 0.00	24.08 ± 0.03	4.98 ± 0.00	0.46 ± 0.01	3.99 ± 0.00	0.34 ± 0.00	2.45 ± 0.08
CREA	104.73 ± 0.00	0.37 ± 0.00	40.67 ± 0.00	54.47 ± 0.46	0.12 ± 0.00	0.01 ± 0.00	0.27 ± 0.01	0.02 ± 0.00	2.31 ± 0.02
CFR	130.08 ± 0.11	0.56 ± 0.00	48.47 ± 0.08	101.64 ± 1.41	0.15 ± 0.01	0.05 ± 0.01	0.05 ± 0.00	0.01 ± 0.00	0.37 ± 0.00

^a^ Mean ± *SD*; *n* = 3.

### 3.4 Simultaneous determination of six bioactive components by HPLC–PDA

**FIGURE 3:**
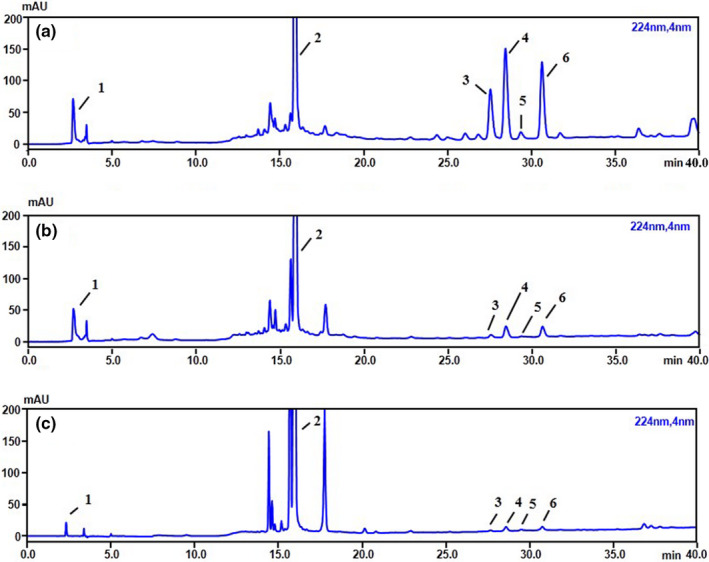
High‐performance liquid chromatography–photodiode array detection (HPLC–PDA) chromatograms of Citri Exocarpium Rubrum (CER) (A), Citri Reticulatae Endocarpium Alba (CREA) (B), and Citri Fructus Retinervus (CFR) (C). 1. Synephrine. 2. Hesperidin. 3. Nobiletin. 4. 3, 5, 6, 7, 8, 3', 4'‐heptamethoxyflavone (HMF). 5. Tangeretin. 6. 5‐hydroxy‐6,7,8,3’,4’‐pentamethoxyflavone (5‐HPMF)

Good linear correlations and the relative standard deviations (RSDs) of repeatability (0.671%–2.595%), precision (0.463%–2.953%), and stability (1.110%–2.449%) were obtained and the recovery was within 97.541%–102.372%, indicating that the HPLC–PDA method is reliable and suitable for CER, CREA, and CFR analyses. The HPLC–PDA chromatograms are shown in Figure 3.

As observed in Table 3, CER, CREA, and CFR have a high content of hesperidin, of which CFR (101.64 ± 1.41 mg g^−1^) had 1–2 times higher concentration of hesperidin than CREA (54.48 ± 0.46 mg g^−1^), followed by CER (24.08 ± 0.03 mg g^−1^). The content of four PMFs (nobiletin, HMF, tangeretin, and 5‐HPMF) in CER was much higher than in CREA and CFR, which was consistent with the results of the TPMFC determined in Section 3.3. Besides, CER and CREA had a higher concentration of synephrine (2.45 ± 0.08 mg g^−1^ and 2.31 ± 0.02 mg g^−1^, respectively) than CFR (0.37 ± 0.00 mg g^−1^).

## 4 DISCUSSION

The work found that CER was highest abundant in volatile components (D‐limonene, 71.71%; γ‐terpinene, 11.97%) and pharmacological research has demonstrated that D‐limonene exhibited anti‐inflammatory activity in the prevention and control of respiratory injuries (Santana et al., 2020). Besides, CER was also rich in PMFs (nobiletin, HMF, tangeretin, and 5‐HPMF) that are mainly found in citrus. PMFs have been reported to show pharmacological effects on the anti‐inflammatory activity (Duan et al., 2017), and a study has mentioned that nobiletin inhibits growth and induces apoptosis in human nasopharyngeal carcinoma (Zheng, Hu, et al., 2019). CER is mainly used for eliminating dampness and phlegm, and other respiratory diseases in the clinical application of Chinese medicine, which may be closely related to its abundant volatile components and PMFs. It was reported that hesperidin exhibits various biological activities in insulin‐sensitizing, antioxidant, lipid‐lowering (Li and Schluesener, 2017). Besides, synephrine also potentially inhibits the conversion of carbohydrates to lipids (Maldonado et al., 2018). Hesperidin and synephrine can enhance cell energy metabolism, and CREA was abundant in both these compounds, which may be the reason that CREA is employed to improve digestion ability in clinical application. Among the three herbs, CFR was extremely rich in hesperidin, TFC, and TPC. Phenolic compounds, including flavonoids, have antioxidant properties that have favorable effects on thrombosis (Kris‐Etherton et al., 2002). It is a possible explanation that CFR is traditionally used to dredge meridian and promote blood flow in clinical application due to its plentiful phenolic compounds.

In addition, natural medicinal herbs have various bioactive chemical components. Relative contents of different bioactive chemical components also lead to different pharmacological activities. For instance, a previous study has mentioned that the relative contents of synephrine and nobiletin coexisted in citrus, significantly affecting the vasoconstriction pattern since synephrine and nobiletin competitively blocked or activated the same contractile targets (Kim et al., 2019). Therefore, the differences in the phytochemicals of CER, CREA, and CFR were further clarified in this study, which might provide the chemical basis for explaining the different pharmacological activities and rational development of them.

## 5 CONCLUSION

**FIGURE 4:**
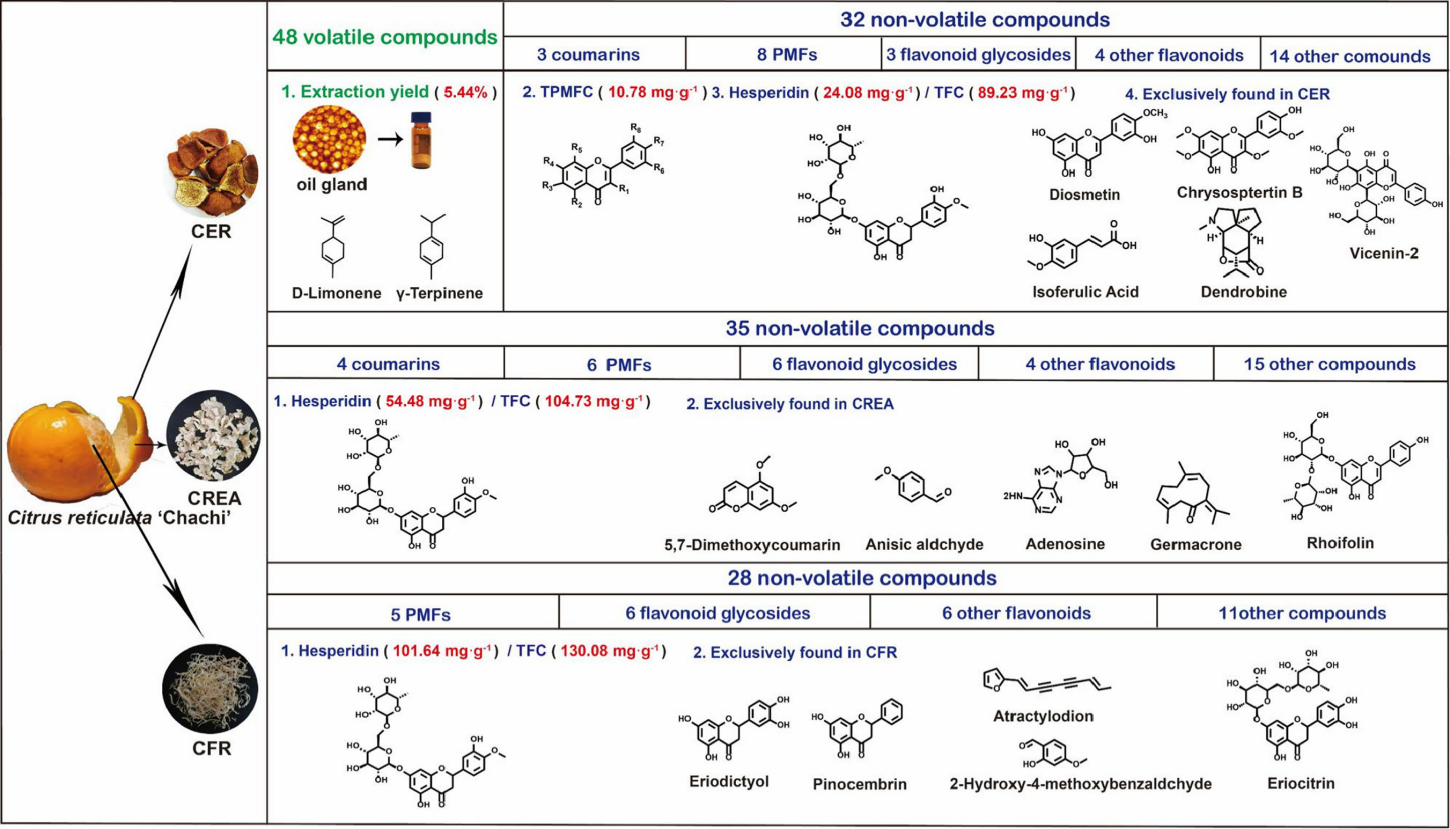
The representative chemical profiling of Citri Exocarpium Rubrum (CER), Citri Reticulatae Endocarpium Alba (CREA), and Citri Fructus Retinervus (CFR)

The volatile components of the dried peel of *Citrus reticulata* “Chachi” were mainly distributed in the CER, the red outer peel containing oil gland, which includes D‐limonene (71.71%), γ‐terpinene (11.97%), α‐pinene (2.71%), and others. Simultaneously, a total of 47 nonvolatile constituents were isolated and identified from the three herbs using UHPLC–Q‐Exactive Orbitrap–MS analysis, among which 32, 35, and 28 nonvolatile constituents were, respectively, identified from CER, CREA, and CFR. Furthermore, CFR had the highest concentration of total flavonoid, total phenolic, and hesperidin, followed by CREA and CER. Conversely, CER was highest abundant in PMFs and the content of nobiletin, HMF, tangeretin, and 5‐HPMF in CREA and CFR was relatively much low. In addition, the content of synephrine in CER and CREA was in the range of 2.00–3.00 mg g^−1^. In summary, the significant differences in important bioactive components of the three TCMs from different parts of fruits peel of *Citrus reticulata* “Chachi” were further clarified in this study, which may provide scientific evidence for comparative research on pharmacological activities and rational application of them (Figure 4).

## CONFLICT OF INTEREST

The authors declare no conflicts of interest.

## Data Availability

The data used to support the findings of this study are included within the article.
